# Construction and Analysis of Survival-Associated Competing Endogenous RNA Network in Lung Adenocarcinoma

**DOI:** 10.1155/2021/4093426

**Published:** 2021-02-11

**Authors:** Lixian Chen, Zhonglu Ren, Yongming Cai

**Affiliations:** ^1^College of Public Health, Guangdong Pharmaceutical University, Guangzhou 510006, China; ^2^Guangdong Provincial TCM Precision Medicine Big Data Engineering Technology Research Center, Guangzhou 510006, China; ^3^College of Medical Information Engineering, Guangdong Pharmaceutical University, Guangzhou 510006, China

## Abstract

Increasing evidence has shown that noncoding RNAs play significant roles in the initiation, progression, and metastasis of tumours via participating in competing endogenous RNA (ceRNA) networks. However, the survival-associated ceRNA in lung adenocarcinoma (LUAD) remains poorly understood. In this study, we aimed to investigate the regulatory mechanisms underlying ceRNA in LUAD to identify novel prognostic factors. mRNA, lncRNA, and miRNA sequencing data obtained from the GDC data portal were utilized to identify differentially expressed (DE) RNAs. Survival-related RNAs were recognized using univariate Kaplan-Meier survival analysis. We performed functional enrichment analysis of survival-related mRNAs using the clusterProfiler package of R and STRING. lncRNA-miRNA and miRNA-mRNA interactions were predicted based on miRcode, Starbase, and miRanda. Subsequently, the survival-associated ceRNA network was constructed for LUAD. Multivariate Cox regression analysis was used to identify prognostic factors. Finally, we acquired 15 DE miRNAs, 49 DE lncRNAs, and 843 DE mRNAs associated with significant overall survival. Functional enrichment analysis indicated that survival-related DE mRNAs were enriched in cell cycle. The survival-associated lncRNA-miRNA-mRNA ceRNA network was constructed using five miRNAs, 49 mRNAs, and 21 lncRNAs. Furthermore, seven hub RNAs (LINC01936, miR-20a-5p, miR-31-5p, *TNS1*, *TGFBR2*, *SMAD7*, and *NEDD4L*) were identified based on the ceRNA network. LINC01936 and miR-31-5p were found to be significant using the multifactorial Cox regression model. In conclusion, we successfully constructed a survival-related lncRNA-miRNA-mRNA ceRNA regulatory network in LUAD and identified seven hub RNAs, which provide novel insights into the regulatory molecular mechanisms associated with survival of LUAD, and identified two independent prognostic predictors for LUAD.

## 1. Introduction

Lung cancer is the most commonly diagnosed and lethal malignancy worldwide [1]. Lung adenocarcinoma (LUAD) is a common subtype of lung cancer [2]. Despite the recent advances in targeted therapeutic strategies, the outcomes of the available treatment strategies for LUAD remain unsatisfactory owing to the drug resistance and relapse, and the five-year overall survival is less than 20% [3]. Therefore, there is an urgent need to understand the molecular mechanisms underlying the pathogenesis of LUAD and identify novel potential prognostic biomarkers to improve prognosis of the disease.

Genetic mutations and dysregulation that can contribute to the pathogenesis of cancer are served as biomarkers. Mutations in epidermal growth factor receptor (*EGFR*) occur in approximately 20% cases of lung cancer [4], and epidermal growth factor receptor-tyrosine kinase inhibitors (EGFR-TKIs) are indispensable in the treatment of EGFR-mutant advanced LUAD. Next-generation sequencing technology has been used to study the role of various RNAs in greater depth. Long noncoding RNAs (lncRNAs) have been considered as potential biomarkers and therapeutic targets due to their unique expression in various cells [5]. Several studies have indicated that dysregulation of lncRNAs, such as MIR31HG [6] and LINC01512 [7], promotes the progression and proliferation of tumour cells in LUAD. microRNAs (miRNAs) play a crucial role in the regulation of protein expression and therefore are considered potential biomarkers in cancer diagnosis. Wang et al. [8] identified a four-miRNA signature comprising miR-142-5p, miR-409-3p, miR-223-3p, and miR-146a-5p, for an early detection of LUAD. Xu et al. [9] reported that miRNA-21, miRNA-125b, and miRNA-224 are associated with chemotherapy sensitivity in patients with LUAD. However, the RNA biomarkers require a critical review before their application for clinical decision-making.

The competing endogenous RNA (ceRNA) network hypothesis, which states that noncoding RNAs (ncRNAs), miRNAs, and mRNAs communicate with each other through microRNA response elements (MREs), has been implicated in posttranscriptional regulation [10, 11]. miRNAs repress the translation of target mRNAs by partial or complete complementary binding to MREs on their target RNA transcripts [12, 13]. ncRNAs can act as endogenous miRNA sponges to competitively bind miRNAs through shared MREs to regulate the expression levels of mRNAs, thereby forming specific ceRNA regulatory network comprising ncRNA-miRNA-mRNA interactions [14]. In the past decade, the study of ceRNAs has gained increased attention and several studies have reported their involvement in tumorigenesis [15], migration [16], and prognosis [17]. For example, lncRNA MAFG-AS1 regulates the expression of *MAFG* to facilitate proliferation of LUAD cells via miR-744-5p [18]. The ceRNA hypothesis provides novel insights into tumorigenesis [19] and biomarker identification [11] at the system biology level.

Bioinformatics techniques are used to integrate and analyse large-scale genomic data, such as RNA-Seq and microarray, to discover potential molecular mechanisms and identify biomarkers, and to guide further experiments. Kumar et al. identified hub genes as potential biomarkers from a large number of differentially expressed (DE) genes by protein-protein interaction (PPI) network [20] and enrichment analysis [21, 22], providing valuable ideas for further study. Wan et al. [23] identified a prognosis-associated ceRNA axes in prostate cancer based on RNA sequencing data using bioinformatics approaches, and validated their regulatory mechanisms by cell proliferation and dual luciferase reporter assay.

In this study, we aimed to construct a ceRNA network associated with survival in DE genes to reveal the molecular mechanisms underlying LUAD and initially identify prognostic factors, thereby to provide new ideas for further biological experiments. In addition to identifying the relationship among various RNAs based on the RNA interaction database, we also performed three statistical tests on lncRNA-mRNA pairs to screen for significant ceRNA interactions based on the ceRNA network hypothesis. The concise LUAD ceRNA network proposed by us would provide accurate and reliable results for subsequent studies.

## 2. Materials and Methods

### 2.1. Data Source and Preprocessing

The GDC data portal (https://portal.gdc.cancer.gov/) [24] is an accessible high-quality cancer genome data-sharing platform that provides primary processed genomic data (level 3 data). We acquired level 3 RNA-Seq (including mRNA and lncRNA) and miRNA-Seq RNA expression data (HTSeq-counts), and clinical information of LUAD patients, who were part of TCGA project from GDC on October 15, 2019. After excluding duplicate samples and other tissue samples, mRNA and lncRNA dataset included 524 cancer samples and 59 adjacent nontumour tissue samples and isoform quantification data from miRNA-Seq included 516 cancer samples and 46 adjacent nontumour tissue samples.

**Figure 1:**
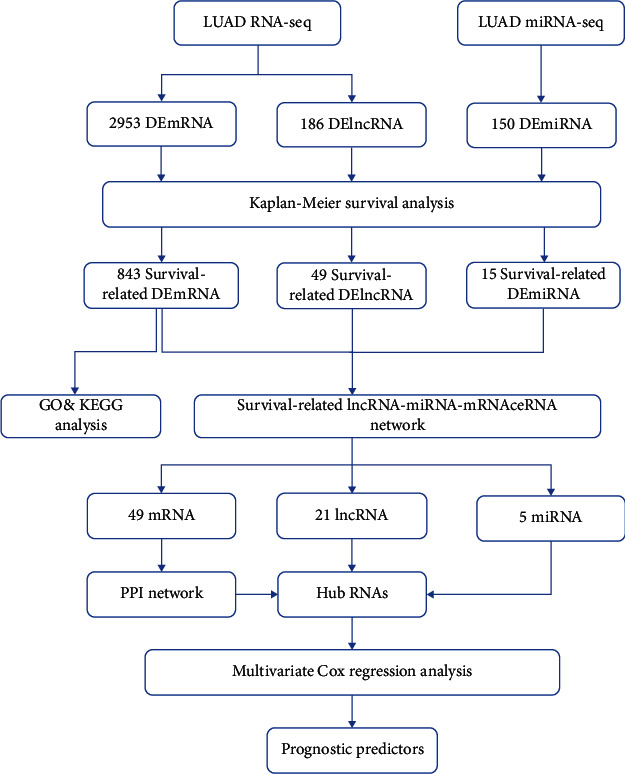
Flow chart of ceRNA network construction.

The RNA-Seq and miRNA-Seq data were processed using the R package GDCRNATools [25]. The raw RNA counts were normalised using the trimmed mean of *M* value (TMM) method [26] and transformed via the voom method [27], wherein the RNAs with lower expression, where the log CPM was found to be lower than 1 in more than half of the samples, were filtered out. The procedure followed in this study is demonstrated in Figure 1.

### 2.2. Differential Expression Analysis

The limma [28] method was used to identify DE RNAs. Fold change (FC) refers to the differences in RNA expression within samples, and the ∣FC | >2 as the threshold value was set based on previous studies on the ceRNA network [29, 30]. ∣FC | >2 and false discovery rate (FDR) < 0.01 were considered statistically significant. Compared to adjacent nontumour tissue samples, RNAs with a higher expression level (FC > 2) in tumour tissue samples were considered upregulated DE RNAs, whereas RNAs with lower expression level (FC < −2) were considered downregulated DE RNAs.

### 2.3. Survival Analysis of DE RNAs

Univariate Kaplan-Meier survival analysis was performed to determine the correlation between the expression level of each DE RNA and the survival time of patients with LUAD. LUAD patients were categorised into high- and low-expression groups based on the median expression of certain DE RNAs. The hazard ratio (HR) of the two groups was evaluated using the Kaplan-Meier plot, and their difference was assessed by performing the log-rank test using the survival package of R [31]. Results with *p* < 0.05 were considered statistically significant.

### 2.4. Functional Enrichment Analysis

The clusterProfiler package [32] of R software is a widely used method for functional enrichment analysis. This package performs overrepresentation and hypergeometric tests to identify DE mRNAs enriched in biological functions or processes. Several enrichment methods ignore the numerical information of DE mRNAs. However, the STRING database (https://string-db.org/) [33] provides another platform to analyse the numerical data via the two-sided Kolmogorov-Smirnov test and aggregate fold change test that perform well in various settings [33, 34]. We used aforementioned two tools to perform functional enrichment analysis on survival-related DE mRNAs and their FC value (for STRING). The cut-off value was set as *p* adjusted < 0.01 for clusterProfiler and FDR < 0.01 for STRING.

### 2.5. Construction of Survival-Associated ceRNA Networks and Identification of Prognostic Predictors

We used miRcode (http://www.mircode.org/) [35] to predict the potential interactions between survival-related miRNAs and lncRNAs. Starbase (http://starbase.sysu.edu.cn/) [36] and miRanda (http://www.microrna.org/microrna/home.do) [37] were used to predict target genes of survival-related miRNAs. Starbase uses multiple algorithms and Ago-binding sites to predict miRNA target sites and their target genes. miRanda predicts the miRNA-mRNA interactions with optimal sequence complementarity using a weighted dynamic programming algorithm and thermodynamic analysis. Therefore, we chose these two databases to improve the reliability of the prediction outcomes.

The ceRNA hypothesis proposed that lncRNAs and their target mRNAs had a positive correlation and they shared miRNAs. Therefore, the competing endogenous interactions between lncRNA and mRNA were evaluated by performing three different statistical tests using GDCRNATools package to select ceRNA pairs matching the ceRNA hypothesis. First, a hypergeometric test was performed to test whether lncRNA and mRNA significantly share a number of miRNAs. Second, Pearson correlation analysis was performed to test the positive correlation between lncRNA and mRNA expressions. Third, regulation pattern analysis [38] was used to measure the regulatory role of miRNAs on lncRNAs and mRNAs. The test criteria were *p* < 0.05, and regulation similarity was not equal to 0.

The lncRNA-miRNA-mRNA ceRNA regulatory network associated with the survival of LUAD was constructed using Cytoscape 3.7.1 [39]. Hub nodes of the ceRNA network were identified using Cytoscape plugin cytoHubba [40]. We imported the mRNAs from the ceRNA network into the STRING database [33] and selected “Homo sapiens” in organism and medium confidence in the minimum required interaction score to obtain a PPI network. The hub RNAs were subjected to multivariate Cox regression analysis to identify independent prognostic predictors.

## 3. Results and Discussion

### 3.1. Survival-Related DE RNAs

**Figure 2:**
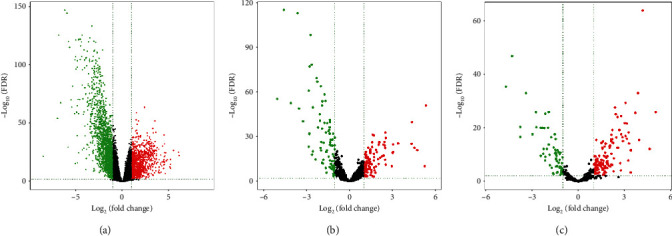
Volcano plots of the DE RNAs: (a) DE mRNAs, (b) DE lncRNAs, and (c) DE miRNAs. The green dots indicated downregulated DE RNAs and the red indicated upregulated DE RNAs in tumour samples. The black dots indicated excluded RNAs.

Differential expression analysis identified 1097 (37.15%) upregulated and 1856 (62.85%) downregulated DE mRNAs (Figure 2(a)), 104 (55.91%) upregulated and 82 (44.09%) downregulated DE lncRNAs (Figure 2(b)), and 93 (62.00%) upregulated and 57 (38.00%) downregulated DE miRNAs (Figure 2(c)) between tissue samples and adjacent nontumour tissue samples. Heat maps of the three DE RNAs are shown in Figures S1–S3 in the Supplementary Materials. We further analysed the associations between these DE RNAs and survival time using univariate Kaplan-Meier survival analysis. In total, we identified 15 DE miRNAs, 49 DE lncRNAs, and 843 DE mRNAs with significant overall survival in 511 patients with LUAD for subsequent analysis.

### 3.2. Functional Enrichment Analysis of Survival-Related mRNAs

**Figure 3:**
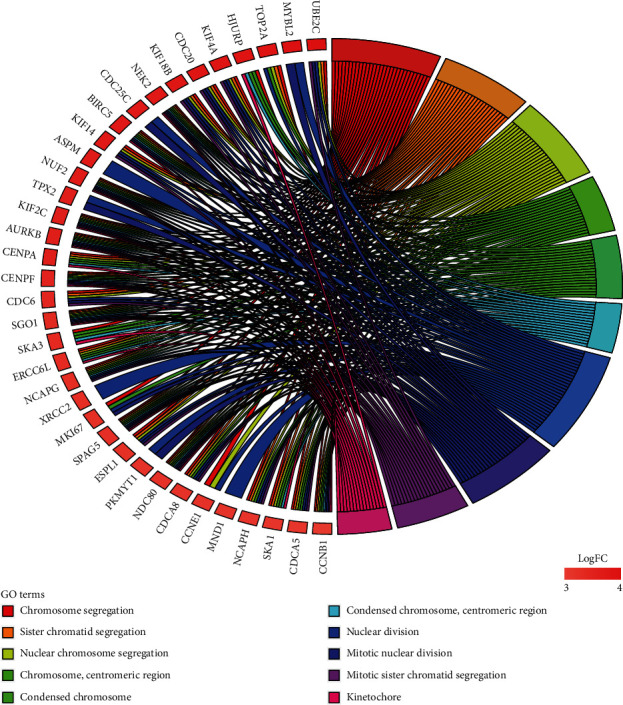
The first 10 GO terms of survival-related DE mRNAs in LUAD from clusterProfiler.

**Figure 4:**
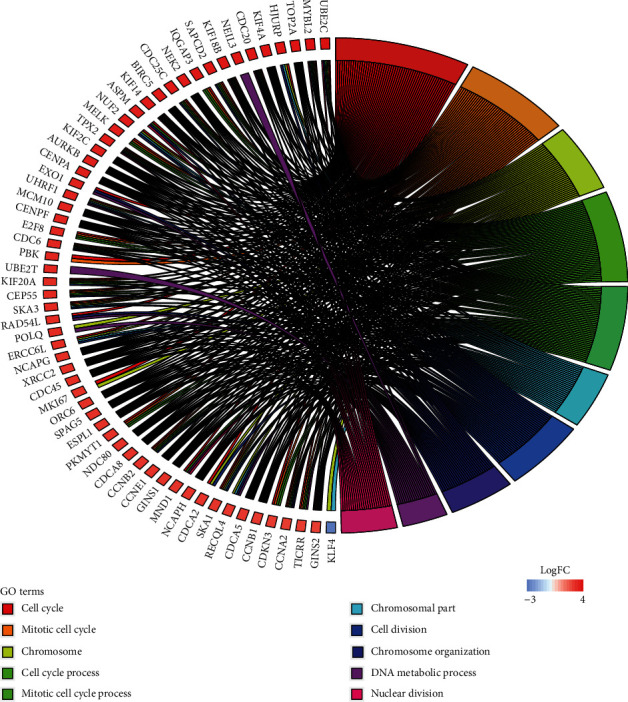
The first 10 terms of survival-related DE mRNAs from STRING.

The 843 mRNAs with significant overall survival were analysed using clusterProfiler and STRING for Gene Ontology (GO) enrichment analysis and identified the first 10 terms with *p* values among three different categories. GO included three different aspects: biological process (BP), cellular component (CC), and molecular function (MF).  Figure 3 shows that 36 upregulated mRNAs with high log_2_FC values were enriched in six terms associated with BP and four cellular components as identified via clusterProfiler. The four terms associated with BP (sister chromatid segregation, nuclear chromosome segregation, mitotic nuclear division, and mitotic sister chromatid segregation) were involved in cell cycle. The four terms associated with CC were involved in chromosome.  Figure 4 shows 60 mRNAs enriched in eight terms associated with BP and the two terms associated with CC as identified by STRING. Similar to the clusterProfiler results, three terms associated with BP involved in cell cycle (mitotic cell cycle, cell cycle process, and mitotic cell cycle process) and two terms associated with CC involved in chromosome. For the MF ontology, the catalytic activity acting on DNA was identified using two different methods.

**Figure 5:**
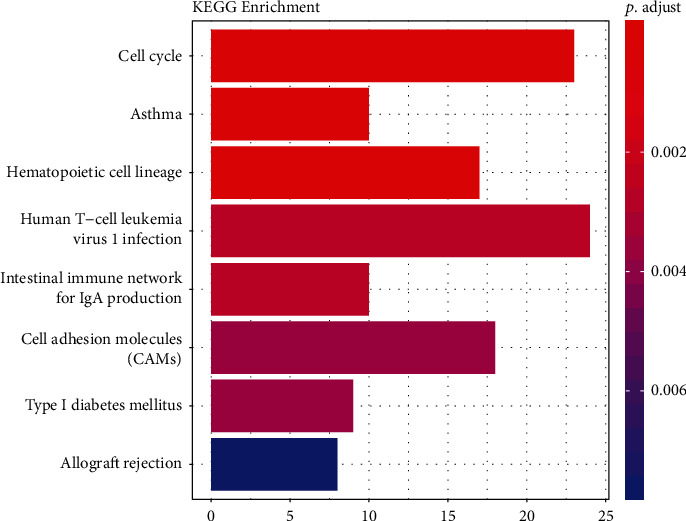
The KEGG pathways of survival-related DE mRNAs in LUAD. The *x*-axis indicates the number of mRNAs participated in the pathway.

The clusterProfiler Kyoto Encyclopedia of Genes and Genomes (KEGG) pathway results (Figure 5) indicated that mRNAs were mainly enriched in cell cycle, human T cell leukaemia virus 1 infection, and cell adhesion molecules (CAMs). STRING identified two different pathways, namely, cell cycle and oocyte meiosis.

The two methods revealed that DE mRNAs were enriched in the cell cycle process and downstream terms. Cell cycle involves progression of cell and nuclear replication and dysregulation of cell replication and division contributes to tumorigenesis. Several studies have reported that overexpression of cell division cycle-associated genes is associated with tumour cell proliferation indicating poor survival in lung cancer patients [41–43]. Rac3 induces apoptosis of LUAD cells via cell cycle pathway and is associated with longer survival [44]. Other KEGG pathways have rarely been mentioned in earlier studies on LUAD. A previous study indicated that human T cell leukaemia virus type I infection induces gene expression of CAMs in lung epithelial cells [45]. However, the association between these pathways and LUAD has not been reported previously.

### 3.3. Survival-Related lncRNA-miRNA-mRNA ceRNA Network in LUAD

**Figure 6:**
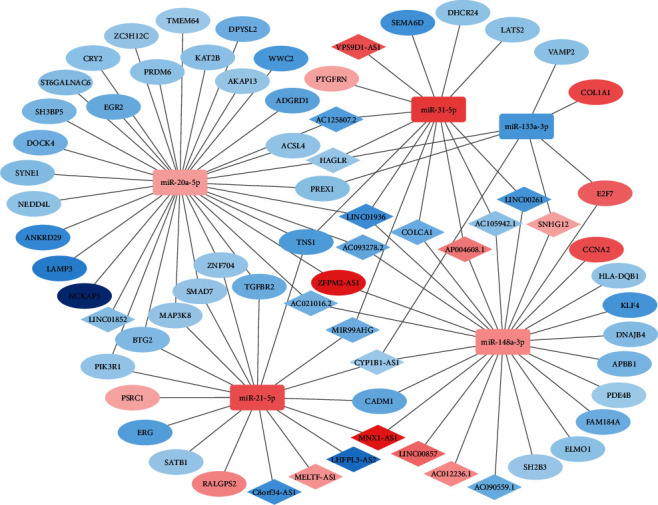
Survival-related lncRNA-miRNA-mRNA ceRNA network of LUAD. The diamonds represent lncRNAs, ellipse mRNAs, and square miRNAs. The red represents upregulated genes, and the blue represents downregulated genes. The shade of the colour represents the magnitude of the value.

To investigate the regulatory interaction in survival-related RNAs, we acquired information on lncRNA-miRNA interactions from miRcode and miRNA-mRNA pairs from Starbase and miRanda. Three statistical tests were performed on lncRNA-mRNA pairs to confirm significant ceRNA pairs. The aforementioned results intersected with survival-significant RNAs. Finally, we considered five miRNAs, 49 mRNAs, and 21 lncRNAs (Table 1) to construct a survival-related ceRNA network (Figure 6) comprising 37 pairs of miRNA-lncRNA interaction and 61 pairs of miRNA-mRNA interaction. This network suggests a potential regulatory relationship between lncRNA-miRNA-mRNA in LUAD prognosis. Several RNAs in the ceRNA network have been verified for their regulatory role in lung cancer or other cancers. LINC00857 regulates cell growth, glycolysis, and apoptosis in LUAD [46]. lncRNA MNX1-AS1 regulates the progression of oesophageal squamous cell carcinoma by targeting the miR-34a/SIRT1 axis [47]. However, most of these interactions have not been previously reported to be associated with LUAD.

The number of mRNAs in the ceRNA network was insignificant to perform functional enrichment analysis via clusterProfiler or STRING. Therefore, we used the PANTHER classification system [48] available on the GO website (http://geneontology.org/). The cellular components and KEGG pathways showed no significant results. All 48 genes, except ZFPM2-AS1, were identified and enriched in 38 terms associated with BP and three terms associated with MF. The results were sorted based on hierarchical relation of terms via PANTHER, with the parent term indented below the subclass (Table 2). Major genes were enriched in the regulation of cellular process (GO:0051244), response to stimulus (GO:0050896), signalling (GO:0023052), and their subclass. The regulation of cellular process involves the regulation of the rate, frequency, and extent of cellular processes. The signalling is a process that transmits information in biological systems. Moreover, the end of signal transduction (GO:0007165) regulates the initiation of transcription [49, 50]. In general, genes in the ceRNA network regulate the activity of various enzymes, participate in signal transduction, and indirectly regulate the initiation of transcription.

### 3.4. Hub RNAs of ceRNA Network and Prognostic Predictor

**Figure 7:**
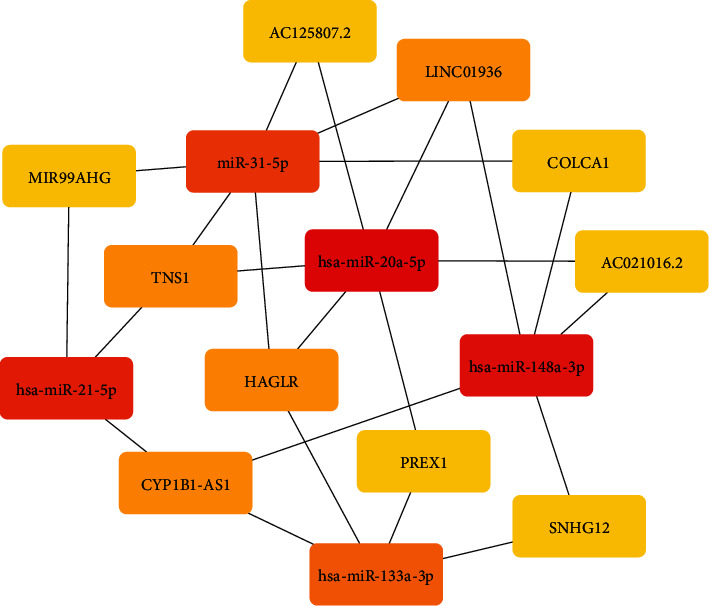
The subnetwork of survival-related ceRNA network. The shade of the colour represents the magnitude of MCC score.

**Figure 8:**
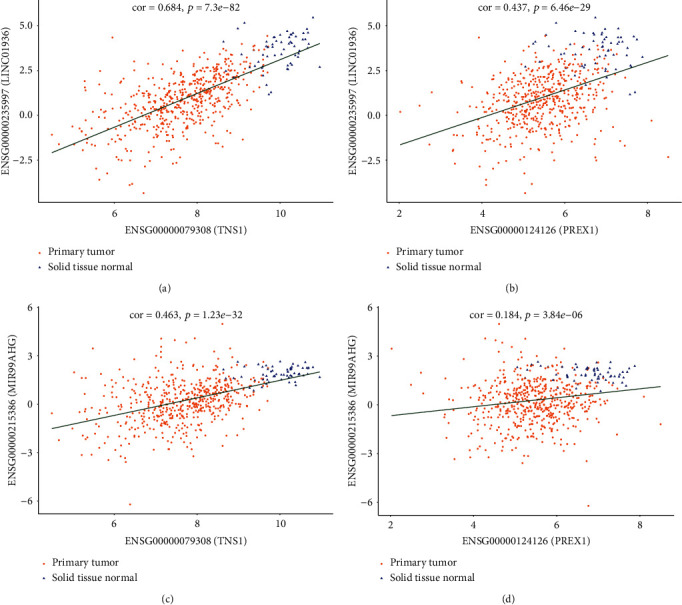
Correlation analysis between lncRNAs and mRNAs of subnetwork: (a) LINC01936 and TNS1, (b) LINC01936 and PREX1, (c) MIR99HG and TNS1, and (d) MIR99HG and PREX1.

**Figure 9:**
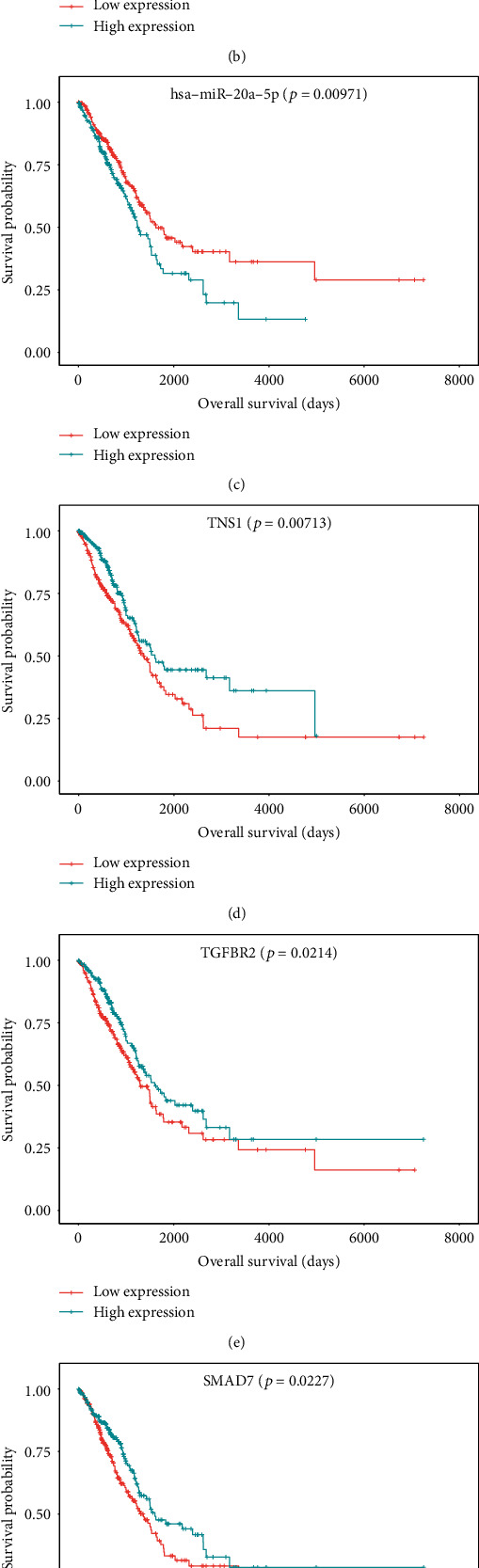
Kaplan-Meier curves of 7 hub RNAs: (a) LINC01936, (b) miR-31-5p, (c) miR-20a-5p, (d) TNS1, (e) TGFBR2, (f) SMAD7, and (g) NEDD4L.

A subnetwork with 15 hub nodes (Figure 7, Table 3) was identified using maximal clique centrality (MCC) in cytoHubba plug-ins. There were a total of six ceRNA pairs in the subnetwork, among which LINC01936-*TNS1* exhibited the highest correlation coefficient (Figure 8) indicating they might have the same expression patterns. LINC01936 and *TNS1* were the highest scoring nodes in their respective categories. The ceRNA network suggested LINC01936 and *TNS1* interacted with miR-20a-5p and miR-31-5p. Our study demonstrated that lower expression of LINC01936 was associated with longer overall survival (Figure 9(a)). However, the role of LINC01936 in LUAD remains unclear. miR-20a-5p exhibited highest topological parameters, indicating that it plays a crucial role in the ceRNA network. Overexpression of miR-20a-5p promotes the migration and invasion of tumour cells [51] and correlates with a shorter survival [52], which is consistent with the findings of our study (Figure 9(c)). The low expression group of miR-31-5p showed better survival (Figure 9(b)). Wei et al. reported that miR31-5p was upregulated in LUAD patients with lymph node metastasis, and low expression of miR-31 was associated with good prognosis in patients with T2N0 stage [53]. *TNS1* participates in fibrillar adhesion formation and cell migration [54] and is involved in signal transduction [55]. However, the role of *TNS1* in tumours remains controversial. *TNS1* negatively regulates tumour migration and invasion, and its high expression is associated with longer metastasis-free survival in breast cancer [56]. Moreover, higher expression of *TNS1* is associated with worse prognosis in colon adenocarcinoma [57]. In this study, we observed that *TNS1* was downregulated in LUAD tissues and that higher expression was associated with better prognosis (Figure 9(d)). Based on our analysis, we predicted that LINC01936 regulates *TNS1* via miR-20a-5p and miR31-5p. However, the role of *TNS1* and its regulatory interaction in LUAD remains unclear and warrants further *in vitro* and *in vivo* studies.

**Figure 10:**
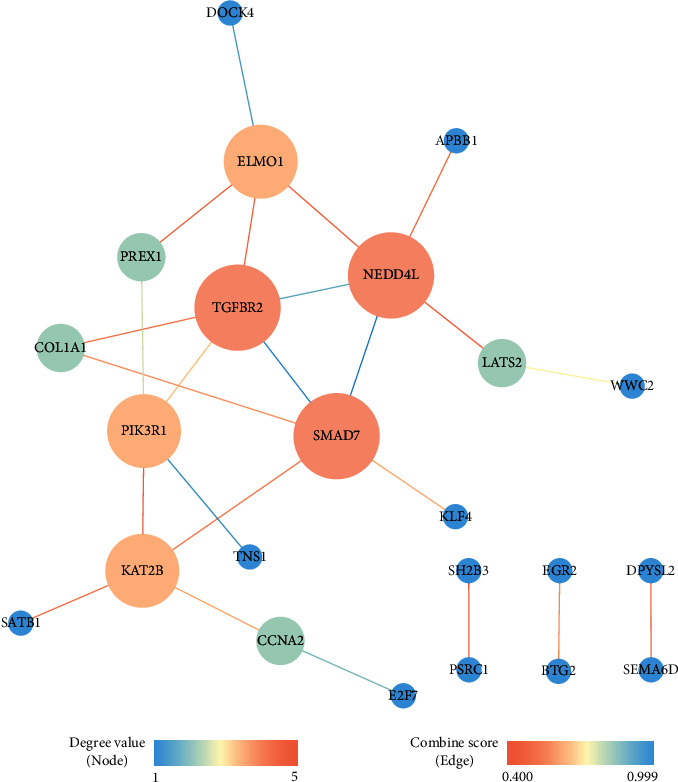
Protein-protein interaction network of survival-related mRNAs in ceRNA network. Size of node represents degree value of mRNA. Line thickness represents the strength of data support (combine score).

The scale of the ceRNA network constructed in this study was small. Some information may have been lost during the identification of hub genes using network topological parameters alone. Therefore, we included genes from the ceRNA network into the STRING to analyse protein interactions to identify hub genes. The PPI network (Figure 10) demonstrated the protein interactions of the ceRNA network. *TGFBR2*, *SMAD7*, and *NEDD4L* with the highest degree were identified as hub genes in the PPI network. *TGFBR2* is the crucial receptor for transforming growth factor-*β* 1 (TGF-*β*1). The TGF-*β*1 ligand binding to *TGFBR2* depends on the serine and threonine residues of the receptor, which in turn binds to the TGF-*β* receptor I to initiate downstream signalling such as Smad and non-Smad signalling pathways to regulate cell proliferation, migration, and apoptosis [58, 59]. *TGFBR2* is downregulated in various cancers [60]. Borczuk et al. reported that low expression of *TGFBR2* associated with lymph node metastasis in patients with LUAD and increased risk of death [61]. Smad complexes translocate to the nucleus to initiate gene transcription. *SMAD7* is an inhibitory Smad molecule that inhibits the formation of Smad complex [62]. Inhibition of miR-21 leads to *SMAD7* upregulation, which inhibits cell invasion via TGF-*β* receptor signalling in non-small-cell lung cancer [63]. A previous study demonstrated that *NEDD4L* can limit TGF-*β* signalling by activating *SMAD2*/*3* [64]. Downregulated *NEDD4L* enhances tumour metastasis and results in poor prognosis [65].

The three hub genes were downregulated in LUAD, and their high expression levels indicated a longer survival (Figures 9(e)–9(g)). Moreover, the ceRNA network showed that LINC01936 is a ceRNA of the three hub genes and mediated through its interaction with miR-20a-5p. In conclusion, several interactions regulate the three hub genes by competitive binding of LINC01936 to miR-20a-5p, which in turn regulate TGF-*β* signalling and downstream signalling pathways. This affects LUAD progression and patient prognosis. To the best of our knowledge, these interactions have not been reported earlier. Thus, our study outcomes lay a strong foundation for future research studies in this field. Moreover, further studies are required to confirm whether silencing or overexpression of LINC01936 affects the expression of hub genes.

Multifactorial Cox regression analysis was performed to identify independent prognostic factors from the abovementioned seven hub RNAs. Our results (Table 4), based on the multifactorial Cox regression model, indicate that LINC01936 and miR-31-5p are independent prognostic predictors of LUAD. LINC01936 was identified as a protective predictor for LUAD, while miR-31-5p was identified as a risk factor. The potential of miR-31-5p as a biomarker has been reported in oral carcinoma [66], colorectal cancer [67], and lung cancer [68]. This study is the first to demonstrate the prognostic potential of LINC01936 in LUAD.

Our study has several limitations. The three RNA expression data and clinical data for this study were based on TCGA database, and our findings lack biological validation. Computational prediction is only a preliminary step in ceRNA research. Therefore, these results need to be verified by studies involving large-scale clinical samples and laboratory methods such as qRT-PCR, luciferase reporter assay, and western blotting. The regulatory mechanism of the ceRNA network needs to be validated by further *in vivo* and *in vitro* research.

## 4. Conclusions

In summary, our study constructed a survival-associated lncRNA-miRNA-mRNA ceRNA network in LUAD using bioinformatics approaches and identified seven hub RNAs (LINC01936, miR-20a-5p, miR-31-5p, *TNS1*, *TGFBR2*, *SMAD7*, and *NEDD4L*). LINC01936 and miR-31-5p were identified as independent prognostic predictors of LUAD. The ceRNA network identified in this study provides novel insights into the molecular regulatory mechanisms associated with LUAD progression. Further studies are required to explore the biological mechanisms of ceRNAs in LUAD and validate the prognostic value of LINC01936 and miR-31-5p in other cohorts.

## Figures and Tables

**Table 1:** The characteristic of RNAs in the survival-related ceRNA network.

Symbol	Differential expression analysis		Kaplan-Meier survival analysis			
	Log_2_FC	FDR	HR	Lower 95	Upper 95	*p* value
miRNA						
miR-31-5p	3.0390	9.65*E* − 12	1.4150	1.0581	1.8923	0.0200
miR-21-5p	2.5664	2.29*E* − 47	1.4347	1.0713	1.9214	0.0133
miR-148a-3p	1.4222	5.86*E* − 19	0.6238	0.4659	0.8352	0.0014
miR-20a-5p	1.1251	2.26*E* − 11	1.4703	1.0965	1.9715	0.0084
miR-133a-3p	-2.5516	1.71*E* − 28	0.7333	0.5484	0.9806	0.0380
lncRNA						
MNX1-AS1	4.3590	2.83*E* − 40	1.3367	1.0005	1.7858	0.0483
VPS9D1-AS1	2.4968	4.97*E* − 23	1.3603	1.0185	1.8169	0.0364
LINC00857	2.0358	4.28*E* − 25	1.4144	1.0594	1.8883	0.0196
AP004608.1	1.7026	9.91*E* − 04	0.7047	0.5278	0.9410	0.0176
AC012236.1	1.4183	4.53*E* − 10	0.6475	0.4847	0.8650	0.0032
MELTF-AS1	1.2753	4.49*E* − 13	1.3454	1.0073	1.7968	0.0438
SNHG12	1.0474	1.98*E* − 10	0.6847	0.5128	0.9143	0.0106
CYP1B1-AS1	-1.0387	8.08*E* − 10	0.7464	0.5586	0.9975	0.0464
HAGLR	-1.1145	1.09*E* − 05	0.7330	0.5490	0.9787	0.0358
AC105942.1	-1.3081	1.45*E* − 28	0.7063	0.5288	0.9433	0.0183
LINC01852	-1.3648	8.65*E* − 37	0.7109	0.5319	0.9500	0.0202
AC021016.2	-1.7220	5.30*E* − 59	0.6566	0.4915	0.8772	0.0043
MIR99AHG	-1.7972	2.33*E* − 26	0.5727	0.4283	0.7658	0.0002
COLCA1	-1.8073	4.92*E* − 15	0.5809	0.4351	0.7757	0.0003
AC090559.1	-1.8911	4.83*E* − 36	0.6959	0.5213	0.9292	0.0152
AC093278.2	-1.9808	1.96*E* − 64	0.7336	0.5495	0.9794	0.0366
AC125807.2	-2.2745	6.44*E* − 70	1.4521	1.0867	1.9403	0.0111
LINC00261	-2.6041	1.66*E* − 18	0.6957	0.5208	0.9293	0.0138
C8orf34-AS1	-2.8281	8.13*E* − 24	0.7072	0.5293	0.9450	0.0184
LINC01936	-2.8318	1.24*E* − 61	0.6948	0.5204	0.9277	0.0141
LHFPL3-AS2	-4.0809	4.86*E* − 53	0.6555	0.4908	0.8754	0.0044
mRNA						
*ZFPM2-AS1*	4.3412	8.62*E* − 26	1.3374	1.0018	1.7855	0.0497
*COL1A1*	2.7177	1.04*E* − 39	1.4556	1.0893	1.9450	0.0104
*CCNA2*	2.5589	2.64*E* − 22	1.7494	1.3091	2.3379	0.0002
*E2F7*	2.2124	1.04*E* − 18	1.6952	1.2665	2.2690	0.0003
*RALGPS2*	1.5246	1.15*E* − 20	1.5304	1.1441	2.0470	0.0036
*PTGFRN*	1.0363	2.55*E* − 18	1.4189	1.0623	1.8951	0.0173
*PSRC1*	1.0255	7.85*E* − 11	1.4873	1.1138	1.9860	0.0075
*PDE4B*	-1.0152	2.67*E* − 14	0.6564	0.4916	0.8764	0.0046
*TMEM64*	-1.0200	3.00*E* − 14	1.3977	1.0460	1.8678	0.0225
*SH2B3*	-1.0734	2.01*E* − 27	0.7333	0.5493	0.9791	0.0371
*AKAP13*	-1.1136	1.52*E* − 19	0.7233	0.5418	0.9657	0.0294
*SATB1*	-1.1413	6.49*E* − 18	0.6896	0.5162	0.9212	0.0117
*MAP3K8*	-1.1418	6.74*E* − 26	0.7273	0.5447	0.9711	0.0313
*ZC3H12C*	-1.1441	5.24*E* − 22	1.5090	1.1294	2.0163	0.0051
*SMAD7*	-1.1465	6.02*E* − 34	0.7137	0.5345	0.9529	0.0227
*PIK3R1*	-1.1601	9.04*E* − 28	0.6737	0.5044	0.8996	0.0076
*ZNF704*	-1.1669	6.09*E* − 13	0.6219	0.4645	0.8326	0.0011
*ACSL4*	-1.1765	3.70*E* − 18	1.3909	1.0409	1.8586	0.0246
*HLA-DQB1*	-1.1923	3.72*E* − 08	0.7161	0.5362	0.9564	0.0237
*DHCR24*	-1.2171	5.25*E* − 22	0.7368	0.5511	0.9851	0.0370
*NEDD4L*	-1.2227	1.94*E* − 20	0.6713	0.5026	0.8964	0.0070
*PRDM6*	-1.2377	3.06*E* − 16	0.7396	0.5539	0.9876	0.0413
*LATS2*	-1.2416	4.69*E* − 46	1.3726	1.0268	1.8348	0.0307
*VAMP2*	-1.2608	9.82*E* − 36	0.7439	0.5571	0.9933	0.0445
*ELMO1*	-1.2697	3.39*E* − 24	0.6072	0.4544	0.8115	0.0007
*CRY2*	-1.2811	1.89*E* − 34	0.6354	0.4759	0.8485	0.0023
*KAT2B*	-1.3000	5.49*E* − 38	0.6358	0.4760	0.8491	0.0022
*PREX1*	-1.3328	1.98*E* − 27	0.7411	0.5550	0.9896	0.0446
*DNAJB4*	-1.4016	1.31*E* − 32	1.5998	1.1981	2.1362	0.0016
*ST6GALNAC6*	-1.4423	1.71*E* − 46	0.6509	0.4873	0.8693	0.0037
*SYNE1*	-1.4435	3.67*E* − 17	0.6758	0.5062	0.9023	0.0088
*BTG2*	-1.4892	1.34*E* − 18	0.6489	0.4858	0.8666	0.0034
*SH3BP5*	-1.5820	1.04*E* − 40	0.7327	0.5487	0.9784	0.0351
*APBB1*	-1.5852	8.53*E* − 39	0.7027	0.5262	0.9385	0.0169
*DOCK4*	-1.7393	7.68*E* − 50	0.7297	0.5466	0.9743	0.0338
*DPYSL2*	-1.7951	2.05*E* − 35	0.6223	0.4659	0.8311	0.0014
*FAM184A*	-1.8098	1.53*E* − 25	0.6544	0.4898	0.8742	0.0040
*TGFBR2*	-1.8527	8.59*E* − 44	0.7118	0.5331	0.9505	0.0214
*EGR2*	-1.9011	5.23*E* − 38	0.7471	0.5596	0.9974	0.0490
*CADM1*	-1.9784	1.95*E* − 21	0.6975	0.5223	0.9316	0.0145
*ADGRD1*	-2.1115	3.18*E* − 21	0.5809	0.4348	0.7762	0.0002
*ERG*	-2.2631	3.57*E* − 83	0.6820	0.5108	0.9106	0.0099
*TNS1*	-2.3744	2.53*E* − 47	0.6708	0.5024	0.8957	0.0071
*WWC2*	-2.4801	2.28*E* − 84	1.4189	1.0609	1.8976	0.0168
*KLF4*	-2.6071	1.81*E* − 45	1.3800	1.0334	1.8428	0.0290
*SEMA6D*	-2.6946	9.92*E* − 61	0.6391	0.4784	0.8536	0.0025
*ANKRD29*	-3.0374	6.21*E* − 50	0.6755	0.5052	0.9031	0.0074
*LAMP3*	-3.4411	1.33*E* − 42	0.7487	0.5607	0.9997	0.0496
*NCKAP5*	-4.4780	1.21*E* − 116	0.6821	0.5109	0.9106	0.0101

FC: fold change; HR: hazard ratio; FDR: false discovery rate.

**Table 2:** GO enrichment analysis of mRNAs in survival-related ceRNA network.

GO terms	Count	FDR
Biological process		
Regulation of cyclin-dependent protein serine/threonine kinase activity	4	4.42*E* − 02
Regulation of cyclin-dependent protein kinase activity	4	4.57*E* − 02
Regulation of cellular process	40	3.16*E* − 02
Biological regulation	42	3.99*E* − 02
Regulation of protein kinase activity	9	4.20*E* − 02
Regulation of kinase activity	11	6.06*E* − 03
Regulation of transferase activity	12	3.64*E* − 03
Regulation of catalytic activity	19	3.22*E* − 03
Regulation of molecular function	22	4.67*E* − 03
Regulation of protein metabolic process	17	4.94*E* − 02
Regulation of developmental growth	7	1.11*E* − 02
Regulation of developmental process	17	3.08*E* − 02
Regulation of growth	10	3.67*E* − 03
Response to peptide hormone	8	4.51*E* − 03
Response to peptide	9	2.89*E* − 03
Response to chemical	26	4.58*E* − 03
Response to stimulus	36	3.70*E* − 03
Response to organonitrogen compound	11	1.12*E* − 02
Response to organic substance	21	2.89*E* − 03
Response to nitrogen compound	11	2.17*E* − 02
Response to hormone	11	3.42*E* − 03
Response to endogenous stimulus	14	3.83*E* − 03
Regulation of cell growth	7	3.11*E* − 02
Negative regulation of catalytic activity	9	4.37*E* − 02
Negative regulation of transcription by RNA polymerase II	10	3.35*E* − 02
Negative regulation of nitrogen compound metabolic process	16	4.36*E* − 02
Negative regulation of protein metabolic process	11	2.51*E* − 02
Intracellular signal transduction	16	2.70*E* − 03
Signal transduction	28	2.62*E* − 03
Signalling	29	2.70*E* − 03
Cell communication	29	2.93*E* − 03
Cellular response to stimulus	30	2.19*E* − 02
Negative regulation of multicellular organismal process	11	4.31*E* − 02
Regulation of multicellular organismal process	20	1.06*E* − 02
Regulation of cell differentiation	15	1.14*E* − 02
Cell differentiation	22	9.65*E* − 03
Cellular developmental process	22	1.17*E* − 02
Positive regulation of biological process	29	1.61*E* − 02
Molecular function		
Guanyl-nucleotide exchange factor activity	6	2.16*E* − 02
Enzyme binding	21	4.54*E* − 05
Kinase binding	10	2.18*E* − 02

FDR: false discovery rate.

**Table 3:** 10 hub RNAs in the ceRNA network ranked by the MCC method.

Rank	Hub RNAs	MCC score	Betweenness centrality	Closeness centrality	Degree
1	miR-20a-5p	34	0.5705	0.4933	34
2	miR-148a-3p	25	0.4053	0.4405	25
3	miR-21-5p	18	0.2492	0.4066	18
4	miR-31-5p	14	0.1858	0.3895	14
5	miR-133a-3p	7	0.0727	0.3627	7
6	LINC01936	3	0.0724	0.4485	3
6	HAGLR	3	0.0507	0.3915	3
6	CYP1B1-AS1	3	0.0482	0.3719	3
6	*TNS1*	3	0.0506	0.4134	3
10	*PREX1*	2	0.0191	0.3507	2
10	MIR99AHG	2	0.016	0.3231	2
10	COLCA1	2	0.0104	0.3348	2
10	AC021016.2	2	0.0414	0.4134	2
10	SNHG12	2	0.0086	0.3203	2
10	AC125807.2	2	0.0206	0.3682	2

MCC: maximal clique centrality.

**Table 4:** Multifactorial Cox regression analysis in hub RNAs.

RNA	*β*	HR	95% CI of HR	*p* value
*TNS1*	-0.02459	0.9757	0.8082-1.1779	0.79806
LINC01936	-0.20487	0.8148	0.7171-0.9257	0.00165
*TGFBR2*	0.15514	1.1678	0.9465-1.4409	0.14790
*SMAD7*	0.10976	1.116	0.8812-1.4134	0.36253
*NEDD4L*	-0.09742	0.9072	0.7643-1.0768	0.26538
miR-20a-5p	0.12756	1.1361	0.9683-1.3329	0.11761
miR-31-5p	0.07502	1.0779	1.0136-1.1462	0.01675

HR: hazard ratio; CI: confidence interval.

## Data Availability

The RNA-Seq and miRNA-Seq data used to support the findings of this study have been deposited in the GDC data portal (https://portal.gdc.cancer.gov/).

## Supplementary Materials

Supplementary Materials Figure S1: heat map of differentially expressed mRNAs. Figure S2: heat map of differentially expressed lncRNAs. Figure S3: heat map of differentially expressed miRNAs.
