# Supplementary figures and images for: Construction and Analysis of Survival-Associated Competing Endogenous RNA Network in Lung Adenocarcinoma
Source: Biomed Res Int. 2021 Feb 11;2021:4093426. doi: 10.1155/2021/4093426 (PMC7895565; doi:10.1155/2021/4093426)

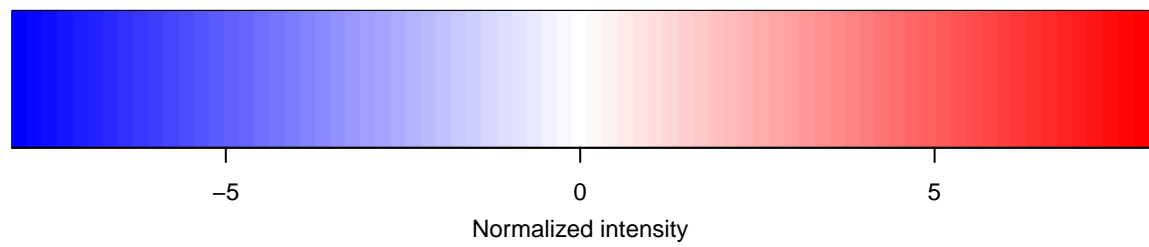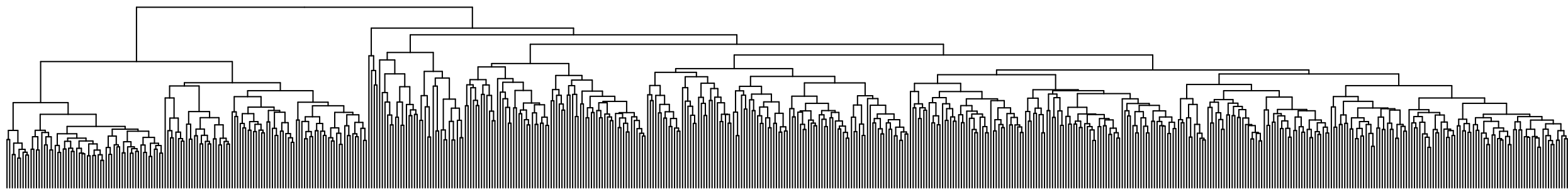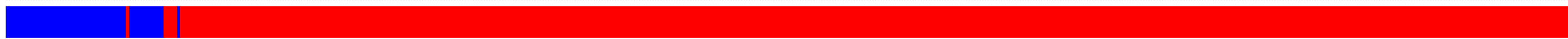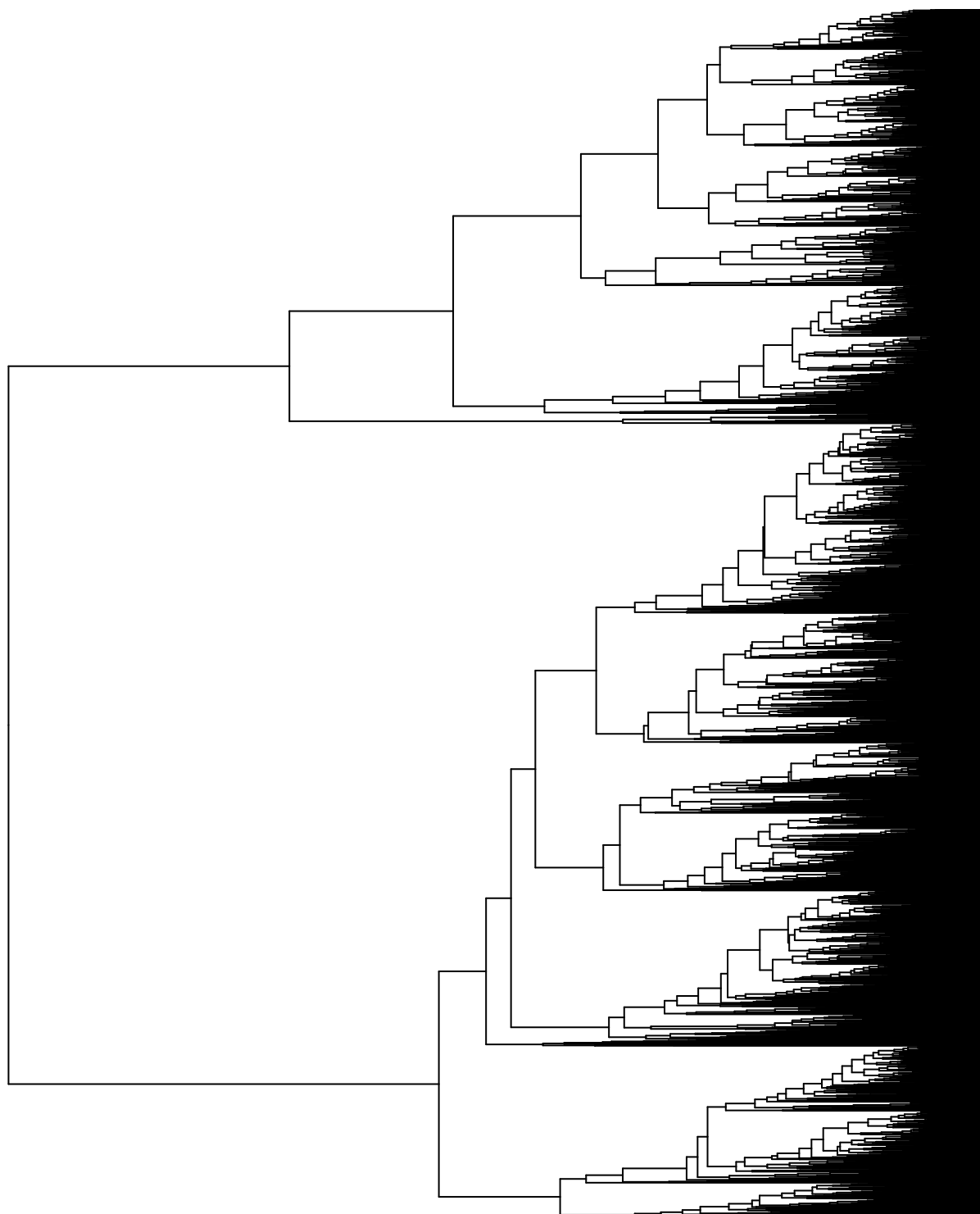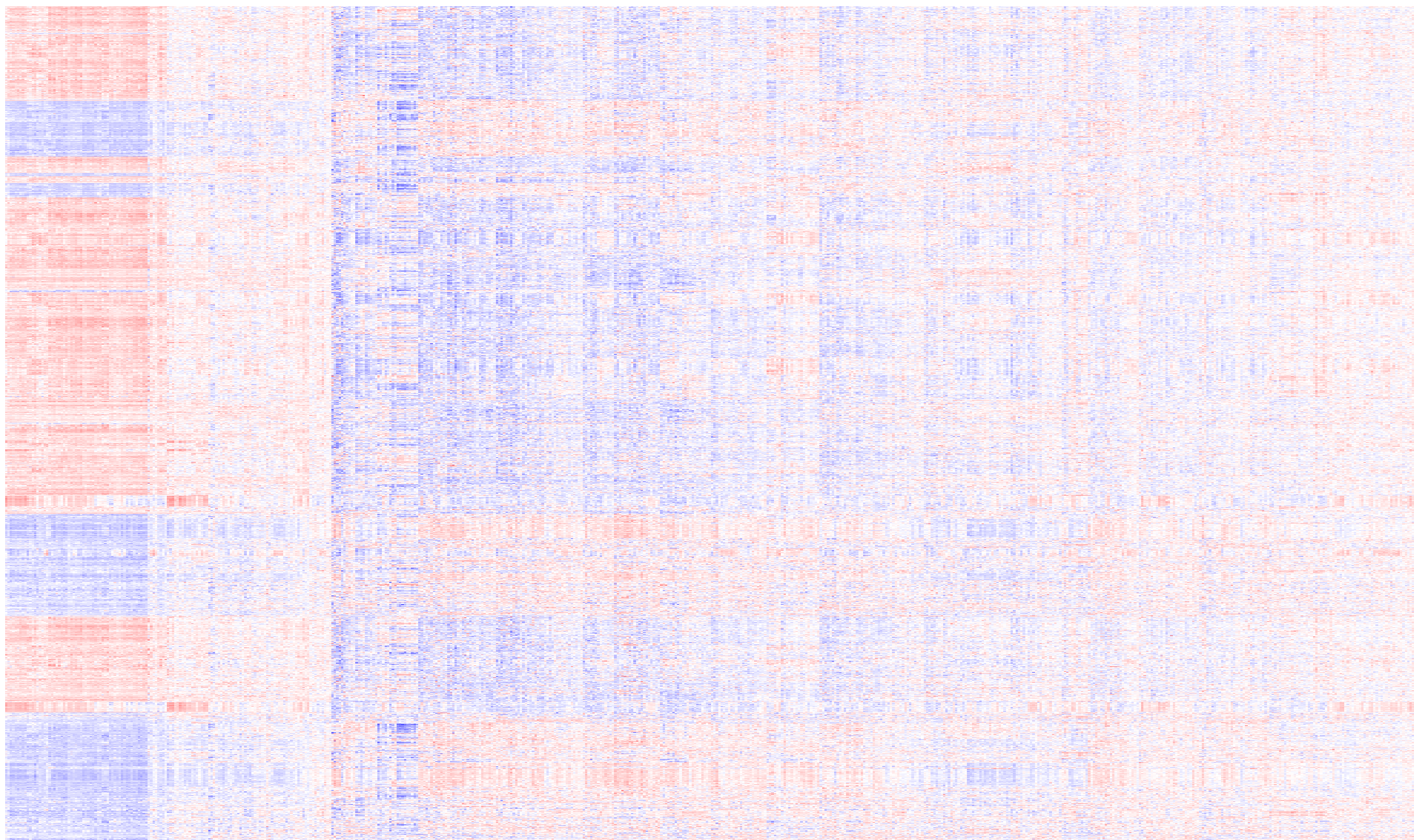

Supplement: Supplementary Materials — Figure S1: heat map of differentially expressed mRNAs. Figure S2: heat map of differentially expressed lncRNAs. Figure S3: heat map of differentially expressed miRNAs. [file 4093426.f1.zip › 4093426.f1/Figure S1 Heat map of differentially expressed mRNAs.pdf]

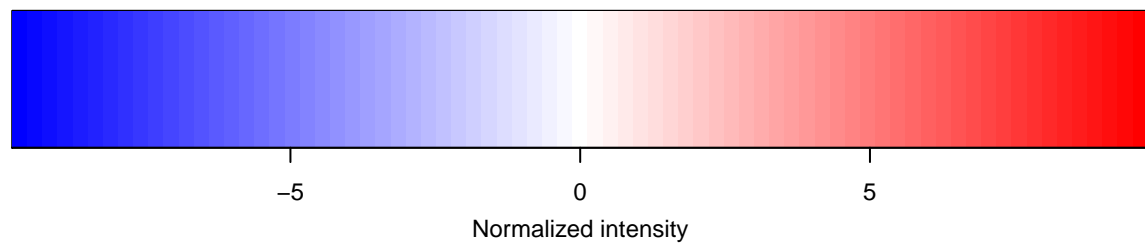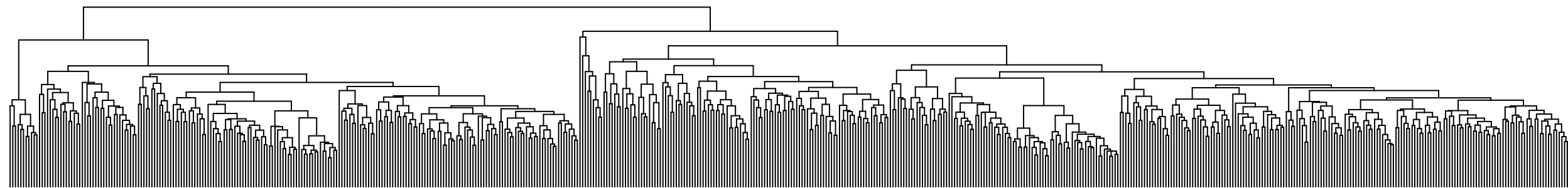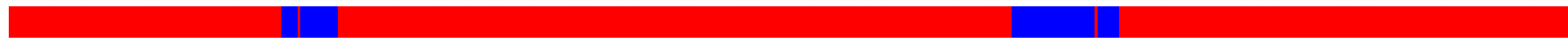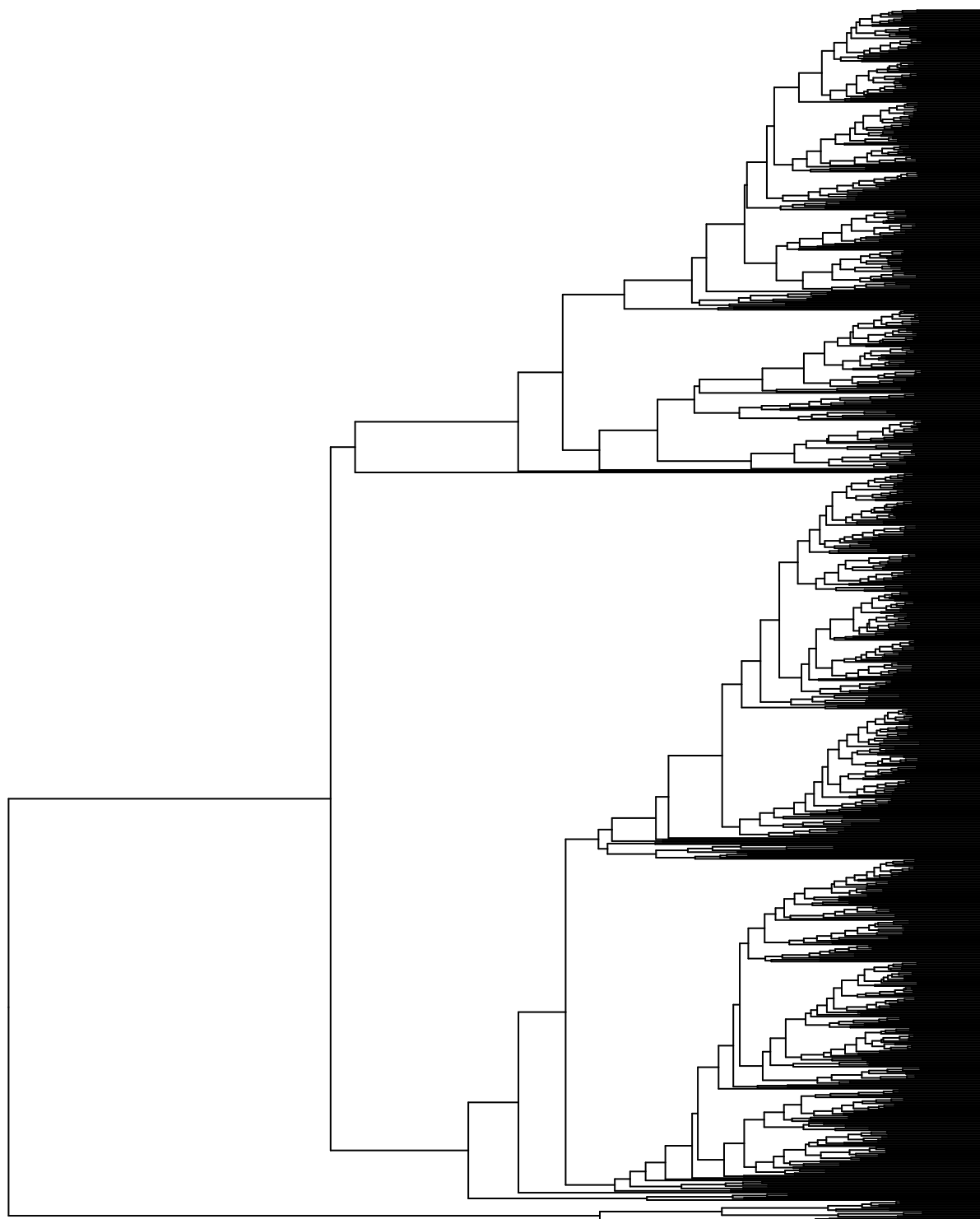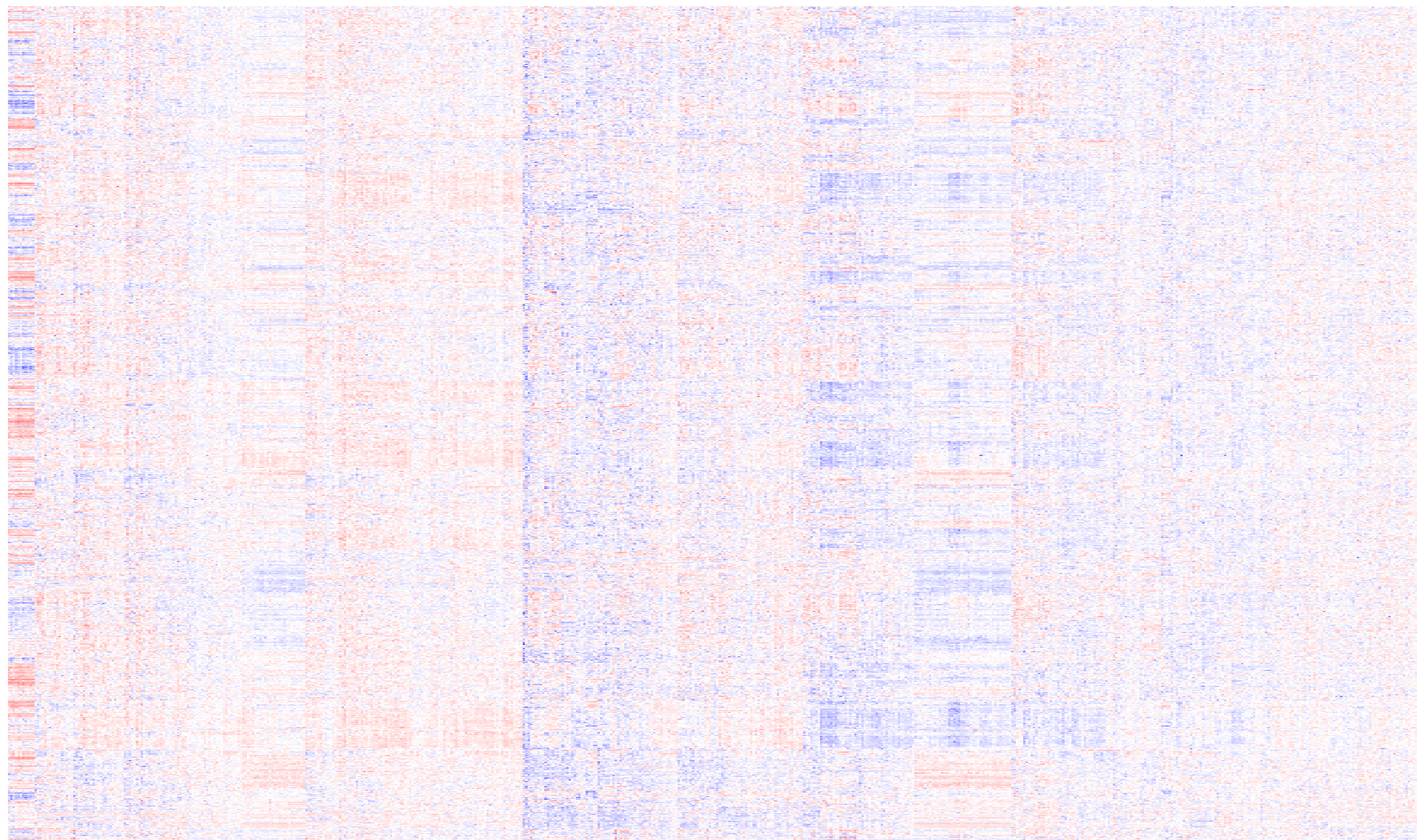

Supplement: Supplementary Materials — Figure S1: heat map of differentially expressed mRNAs. Figure S2: heat map of differentially expressed lncRNAs. Figure S3: heat map of differentially expressed miRNAs. [file 4093426.f1.zip › 4093426.f1/Figure S2 Heat map of differentially expressed lncRNAs.pdf]

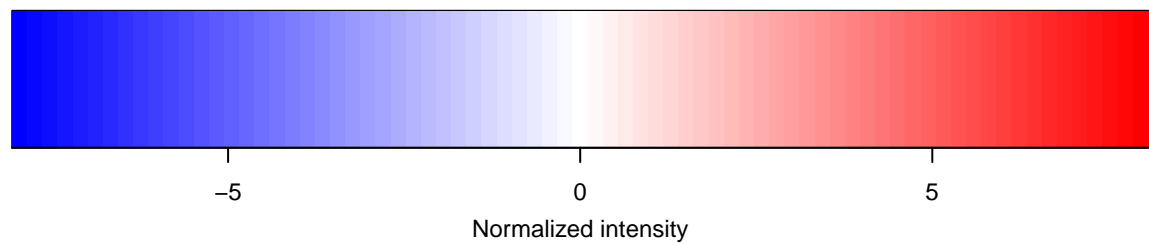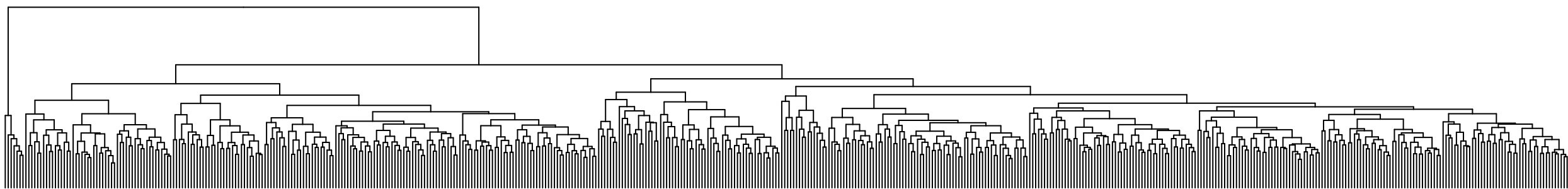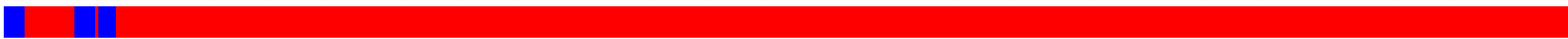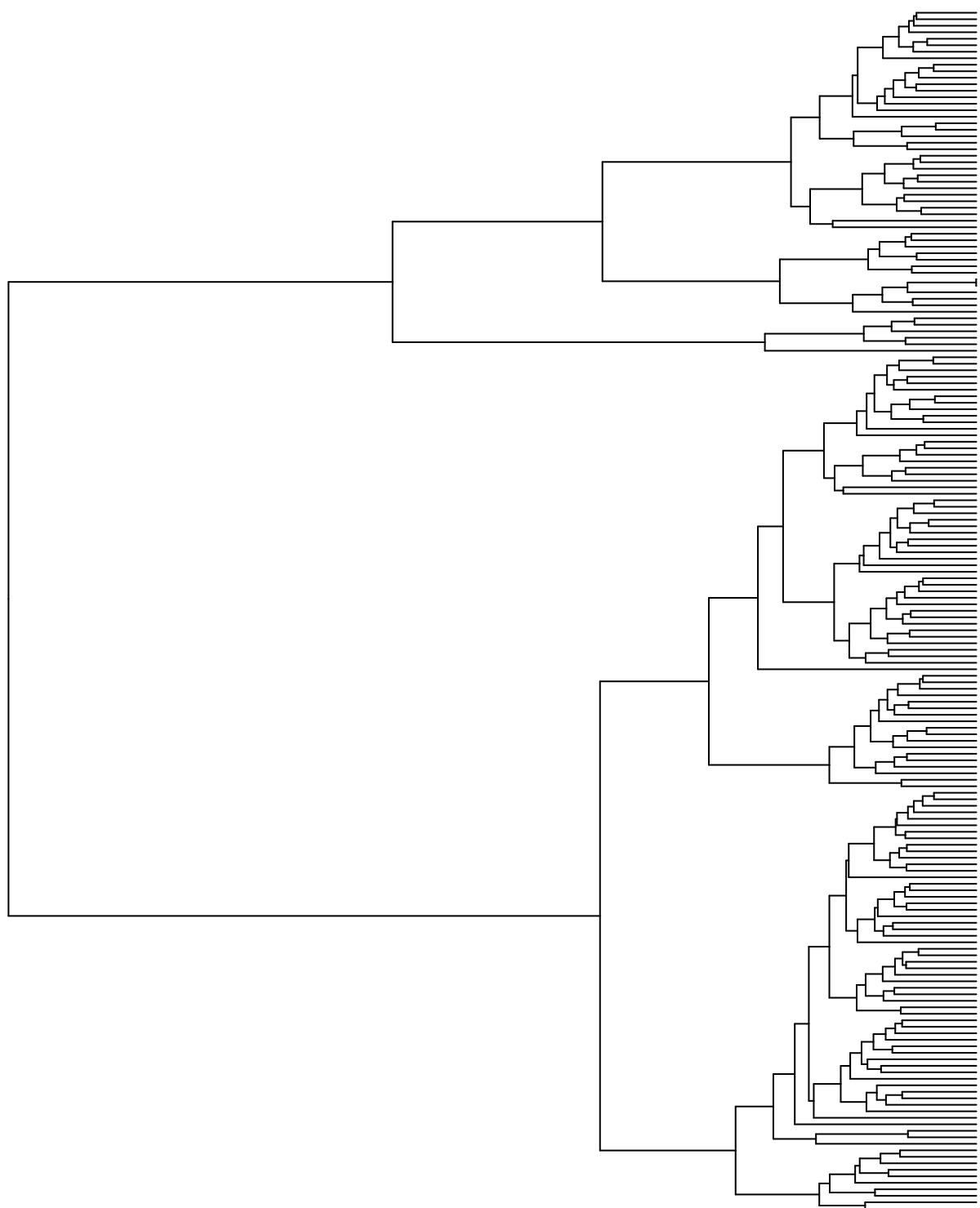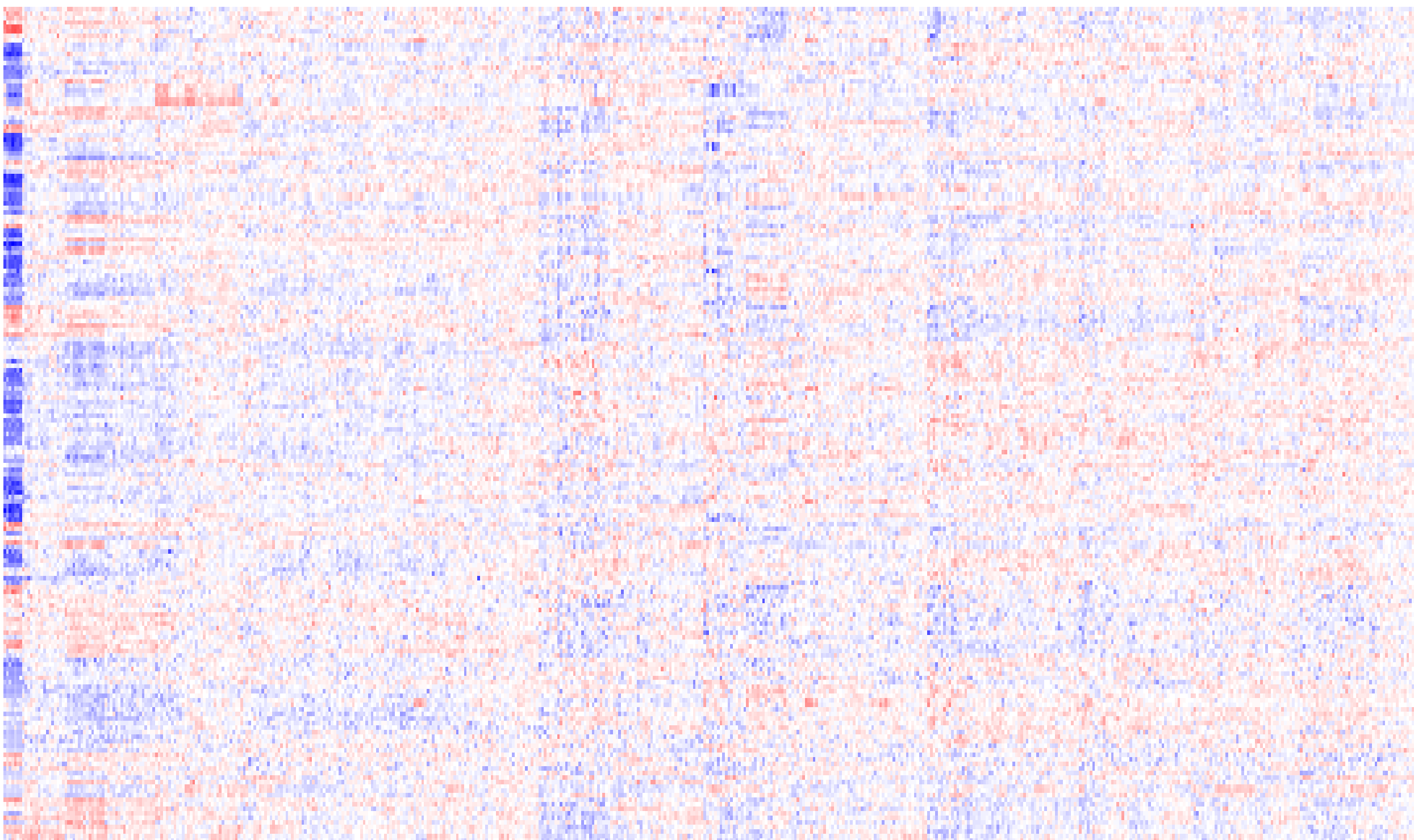

Supplement: Supplementary Materials — Figure S1: heat map of differentially expressed mRNAs. Figure S2: heat map of differentially expressed lncRNAs. Figure S3: heat map of differentially expressed miRNAs. [file 4093426.f1.zip › 4093426.f1/Figure S3 Heat map of differentially expressed miRNAs.pdf]
